# Pristine mangrove creek waters are a sink of nitrous oxide

**DOI:** 10.1038/srep25701

**Published:** 2016-05-12

**Authors:** Damien T. Maher, James Z. Sippo, Douglas R. Tait, Ceylena Holloway, Isaac R. Santos

**Affiliations:** 1 School of Environment, Science and Engineering, Southern Cross University, Lismore, 2480 Australia; 2 National Marine Science Centre, Southern Cross University, PO Box 4321, Coffs Harbour, NSW 2450, Australia

## Abstract

Nitrous oxide (N_2_O) is an important greenhouse gas, but large uncertainties remain in global budgets. Mangroves are thought to be a source of N_2_O to the atmosphere in spite of the limited available data. Here we report high resolution time series observations in pristine Australian mangroves along a broad latitudinal gradient to assess the potential role of mangroves in global N_2_O budgets. Surprisingly, five out of six creeks were under-saturated in dissolved N_2_O, demonstrating mangrove creek waters were a sink for atmospheric N_2_O. Air-water flux estimates showed an uptake of 1.52 ± 0.17 μmol m^−2^ d^−1^, while an independent mass balance revealed an average sink of 1.05 ± 0.59 μmol m^−2^ d^−1^. If these results can be upscaled to the global mangrove area, the N_2_O sink (~2.0 × 10^8^ mol yr^−1^) would offset ~6% of the estimated global riverine N_2_O source. Our observations contrast previous estimates based on soil fluxes or mangrove waters influenced by upstream freshwater inputs. We suggest that the lack of available nitrogen in pristine mangroves favours N_2_O consumption. Widespread and growing coastal eutrophication may change mangrove waters from a sink to a source of N_2_O to the atmosphere, representing a positive feedback to climate change.

Nitrous oxide is a long lived greenhouse gas with an atmospheric lifetime of 118–131 years^1^, a global warming potential about 300 times that of CO_2_, and it is the major contributor to ozone destruction in the stratosphere^2^. Microbial processes within soils and surface waters produce most atmospheric N_2_O through nitrification and denitrification. The largest portion of global denitrification from natural sources is thought to occur within coastal waters (~45%)^3^, with these areas considered globally significant N_2_O sources^4^. However global estimates lack empirical data from some key environments such as mangrove waterways.

Mangrove forests cover about 138,000 km^2^ of coastline and are recognised to contribute disproportionally to global biogeochemical cycles^5^. Mangrove ecosystems are thought to be an important atmospheric N_2_O source^6^,^7^,^8^. Yet, N_2_O observations in mangroves are highly variable and often focus on soil emissions from eutrophic systems influenced by freshwater inputs^9^,^10^. The methodology typically used to estimate soil emissions (i.e. soil chambers) only captures N_2_O fluxes from exposed soils. To date, little work has been done on the rates of N_2_O exchange between surface waters and the atmosphere in mangroves. One study revealed high dissolved N_2_O concentrations, and suggested mangroves were a significant source of N_2_O to the atmosphere^11^. This study was undertaken over two 24-h periods in a mangrove tidal creek influenced by upstream freshwater inputs. However, because rivers are often enriched in N_2_O^12^, upstream riverine inputs of N_2_O may lead to a misinterpretation of the N_2_O source. As a result, it is unclear whether high dissolved N_2_O was related to upstream freshwater inputs or N_2_O production within the mangrove forest.

To understand the role of mangroves within global N_2_O budgets, we investigated dissolved N_2_O dynamics in six pristine mangrove tidal creeks covering a latitudinal gradient in Australia (Fig. 1, Table 1). We initially hypothesised that mangrove waters are a source of N_2_O to the atmosphere, which may play a disproportionately large role in global N_2_O budgets per unit area. To test this hypothesis we used novel, automated, quasi-continuous measurement technology that allowed for high temporal resolution, high precision dissolved N_2_O observations to be made *in situ*. Sites were selected to ensure the N_2_O observations were only related to mangrove sinks and sources. The sites had mangrove dominated catchments and no obvious upstream riverine input or anthropogenic nitrogen sources (Table 1).

High temporal resolution observations over about 5 days in each creek revealed dissolved N_2_O concentrations ranging from 3.4–9.1 nM (50–123% saturation, Table 2, Figs 2 and 3). Nitrate + nitrite (NO_x_) concentrations in all the creeks were low (Fig. 3) and approached oceanic values, which average between 0.04 and 3.52 μM [Table 1, oceanic data was sourced from the Integrated Marine Observing System (IMOS)]. Ammonium (NH_4_) was higher in the two southern sites which also had lower salinity suggesting a freshwater source of NH_4_ to these systems. When data were averaged over the five days of the time series, five out of the six mangrove creeks were under-saturated in dissolved N_2_O, resulting in atmospheric N_2_O uptake by these waters (Fig. 3, Table 2, Supplementary Information). The only creek that was on average supersaturated in N_2_O (Newcastle) had the lowest salinity (Figs 2 and 3, Supplementary Information), suggesting freshwater inputs were the likely source of N_2_O supersaturation, or freshwater supplied the nitrogen fuelling N_2_O production.

The average N_2_O concentrations in each of the six mangrove creeks were lower than in the only previous study measuring dissolved N_2_O over a tidal cycle in mangrove waters which found mean concentrations of 9.0 ± 2.3 nmol L^−1^ (~170% saturation) (dry season) and 8.6 ± 1.3 nmol L^−1^ (~120% saturation) (wet season) over two 24 hour time series in the Andaman Islands^11^. Relatively high dissolved N_2_O concentrations are often found in rivers and inner estuaries, where catchment nitrogen inputs fuel N_2_O production through nitrification and denitrification^13^,^14^,^15^. With salinity ranging from 0 to 28 in the Andaman Island study, the higher N_2_O concentrations observed may have been driven by freshwater nitrogen loads, rather than mangrove related processes.

A complex combination of drivers may influence dissolved N_2_O production and consumption within mangrove ecosystems. Previous studies have found either nitrification^6^,^11^,^16^ or denitrification^7^,^8^,^17^ to be the dominant N_2_O production pathway in mangroves. N_2_O cycling is coupled to NO_x_ and ammonium (NH_4_^+^) availability^17^, and a number of environmental factors such as denitrification and nitrifier bacteria abundance, redox potential, temperature, porewater or groundwater exchange, sulphur cycling, and local conditions such as topography, biomass, species composition and root structure^7^. For example, N_2_O concentration has been found to increase with; increasing denitrifier abundance^18^, increasing temperature^13^ , inputs of groundwater/porewater^19^ or through N_2_O reduction inhibition by H_2_S^20^, and reduced N_2_O production has been linked to low redox potentials in soils^6^.

Pristine mangrove waters generally have low inorganic nitrogen concentrations^21^. Mangroves may conserve nitrogen through nitrate reduction via dissimilatory nitrate reduction to ammonium (DNRA) rather than denitrification^22^. This may enhance the N_2_O sink capacity of mangroves via two ways. First, N_2_O production through denitrification is inhibited due to competition for nitrate by DNRA. Second, DNRA bacteria and archaea possess the enzyme system required to catalyse the reduction of N_2_O to N_2_^23^. Previously it was thought that denitrifiers were the only group capable of N_2_O reduction. In addition, some plants can produce and exude compounds that inhibit nitrification such as cyclic diterpenes^24^. Similar compounds have been found in the bark of mangrove roots^25^. These compounds may act as a chemical defence limiting nitrogen loss from highly productive mangrove ecosystems with limited nitrogen.

Overall, N_2_O atmospheric fluxes for individual systems ranged from −3.43 to 0.71 μmol m^−2^ d^−1^ (mean −1.52 ± 0.17 μmol m^−2^ d^−1^) when using well established flux models (Table 2). Using an independent mass balance approach, mangroves were estimated to have an atmospheric flux of −3.20 to 0.03 μmol m^−2^ d^−1^ (mean −1.05 ± 0.59 μmol m^−2^ d^−1^). Overall, these two independent approaches are consistent in that 5 out of the 6 systems were a sink for atmospheric N_2_O, giving confidence in our observations. The only system that released N_2_O to the atmosphere on average over the five day time series (Newcastle) also had the greatest input of freshwater (Figs 2 and 3).

Upscaling the N_2_O flux from mangrove waters in this study provides a first order estimate of fluxes from pristine mangrove systems. The mean atmospheric flux from the six sites was −1.52 (±0.17) μmol m^−2^ d^−1^ and the global mangrove area is estimated to be 0.36 × 10^6^ km^2 ^11 using the global forested area from Borges *et al*.^26^ (~0.2 × 10^6^ km^2^), and the average wetland forest area from Selvam^27^ (0.16 × 10^6^ km^2^), as done in the only other previous estimate of the global mangrove creek N_2_O flux^11^. The uncertainty associated with this estimate of creek area is unknown, but likely large. Assuming all global mangrove systems are in a pristine state is unrealistic, yet provides a first order estimate of the potential sink capacity of mangrove waters and allows for an update from the only previous estimate based on fluxes measured from one site during two 24 hour measurement campaigns^11^. Further, seasonal variability could not be determined during the current study. Adequate assessment of seasonality in N_2_O fluxes would be required to better constrain these estimates. In spite of these obvious caveats, if our estimates are representative of the N_2_O flux from pristine mangroves, the global N_2_O sink in pristine mangrove systems would be 2 × 10^8^ mol yr^−1^. Previous estimates of N_2_O fluxes from mangrove systems reported a net source to the atmosphere, with global extrapolations of 2.7 × 10^9^ mol yr^−1^ from mangrove soils and waters^11^, and ~3.2 × 10^8^ to 13 × 10^10^ mol N_2_O yr^−1^ from soil emissions alone^10^. In contrast to our investigation, previous studies focused on systems with upstream freshwater inputs and therefore presumably some associated nitrogen inputs, which may fuel N_2_O production via nitrification and denitrification.

The sink nature of N_2_O within pristine mangroves is a rarity for aquatic systems, with most waterbodies being a source of N_2_O to the atmosphere. Estuaries and rivers are thought to contribute approximately 5% of the natural global N_2_O emissions from aquatic systems^28^. If our estimates are representative of mangroves globally, our results imply that pristine mangroves may offset about 3% of estuarine emissions (~7.14 × 10^9^ mols yr^−1^)^28^, or 6% of N_2_O outgassing from rivers (~3.57 × 10^9^ mols yr^−1^)^28^ and therefore play a relatively minor role within the global N_2_O budget from natural waterways. We suggest that the pristine mangrove N_2_O sink behaviour is related to nitrate limitation as well as microbial and plant inhibitory processes (Fig. 4). Combined with previous studies that indicate significant N_2_O releases from mangroves that are higher in nitrate^11^, our observations imply that human induced eutrophication, and in particular increased nitrogen loading may shift mangrove waters from a sink to a source of N_2_O to the atmosphere.

## Methods

**Figure 1:**
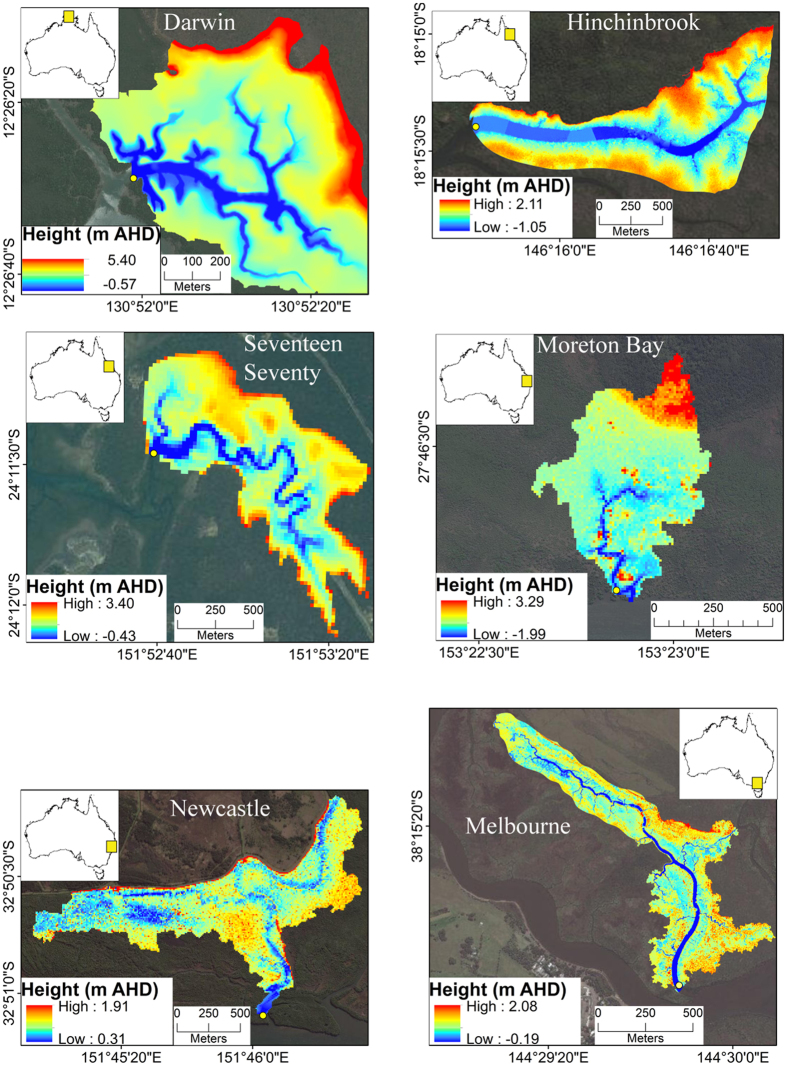
Location of six mangrove creeks on the North and East coast of Australia and the associated digital elevation models (DEM). Map and DEMs created with ESRI ArcGIS version 10.3 (https://www.arcgis.com). Image modified from Tait *et al*. (ref. 35).

This study was undertaken in six pristine mangrove tidal creeks on the Eastern and Northern Australian coast (Fig. 1; Table 1). Importantly, creeks were selected from low lying areas with small catchments in order to prevent significant upstream freshwater inputs that could mask processes occurring within intertidal mangroves. Therefore, freshwater input was assumed to occur only as a result of direct rainfall over the creek and the small intertidal areas.

To measure dissolved N_2_O, creek water was continually pumped from a depth of ~1 m into a showerhead air–water exchanger at a flow rate of approximately 3 L min^−1^. Continuous N_2_O concentrations (±2 ppb) were measured in the shower head exchanger at one second intervals for about five days at each site using a Picarro G2308 cavity ring-down spectrometer (CRDS) as described elsewhere^29^,^30^. The CRDS was calibrated using a 350 ppb standard (Air Liquide, Australia), prior to, and upon completion of the field measurements. Instrument drift was less than 2 ppb between calibrations (i.e. over several weeks). This 2 ppb drift equates to <0.10 nM at *in situ* temperature and salinity (assuming a total 4 ppb error in the concentration difference between the atmospheric and water column measurements). N_2_O dry molar fractions were converted to dissolved N_2_O concentrations as a function of pressure and solubility^31^ (1):


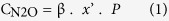


where β is the Bunsen solubility coefficient calculated from temperature and salinity^32^, *x*’ is the dry molar fraction of N_2_O and *P* is ambient pressure. Atmospheric pressure and temperature were measured with a weather station located on site (Davis Vanatge Pro II), and pressure and temperature within the equilibrator was measured with a temperature/pressure logger (Van Essen CTD logger).

At each site, estuarine current velocity and water depth were measured at fifteen minute intervals using an acoustic doppler current profiler (SonTek Argonaut). Salinity, water temperature, pH, and dissolved oxygen were measured at 15 minute intervals using a calibrated multi-parameter water quality sonde (Hydrolab DS5). Dissolved nitrogen concentrations were measured on discrete samples collected hourly during a 24 hour time series (n = 25 per site), and analysed using flow injection analysis (Lachat Quickchem 8000). The samples were filtered with 0.45 μm cellulose acetate filters and kept frozen until analysis within 1 month. NO_x_ detection limits were 0.07 μM (error ±3%) and NH_4_ detection limits were 0.35 μM (error ±5%).

The flux of N_2_O in mangrove waters was estimated using two different approaches. First, we applied a gas exchange model. The transfer of N_2_O between the water and atmosphere was estimated as a function of the concentration in water and atmospheric concentration, solubility and gas transfer velocity at one minute intervals (2):


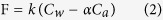


where *k* is the gas transfer velocity, *C*_*w*_ and *C*_*a*_ are the water and air phase concentrations respectively and *α* is the solubility coefficient.

Gas transfer velocity was calculated using an empirical relationship developed specifically for mangroves^33^ (3):


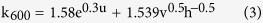


where k_600_ is the gas transfer velocity normalised to a schmidt number of 600, u is the wind speed at a height of 10 m, v is current velocity (cm s^−1^) and h is water depth (m). Positive values indicate a flux of N_2_O from the water to the atmosphere, while negative values indicate gas exchange from the atmosphere to water. Atmospheric concentrations where measured with the same CRDS at least once per day for a period of 5 to 10 minutes. There was no significant difference between the atmospheric dry molar fractions measured at each site (ANOVA p > 0.05), therefore we used the pooled mean of 326 ppb for our atmospheric endmember. Wind speed data was sourced from the Australian Bureau of Meteorology for the nearest station near each site (all stations were within 20 km of the corresponding study site).

Second, we developed a mass balance approach that estimates the net water borne flux of N_2_O in and out of the mangrove catchment. Volumetric water discharge was calculated at one minute intervals using a high resolution LIDAR derived digital elevation model (DEM) with 1 m grid, ±0.1 m elevation accuracy, as described by Maher *et al*.^34^. The net exchange of N_2_O was then calculated as a function of concentration and discharge, with a mass balance constructed incorporating water borne exchange and the net air water flux within the mangroves. The air water flux was calculated as a function of time specific wetted area at one minute intervals, calculated using depth and the DEM.

## Additional Information

**How to cite this article**: Maher, D. T. *et al*. Pristine mangrove creek waters are a sink of nitrous oxide. *Sci. Rep*. **6**, 25701; doi: 10.1038/srep25701 (2016).

## Supplementary Material

Supplementary Information

## Figures and Tables

**Figure 2:**
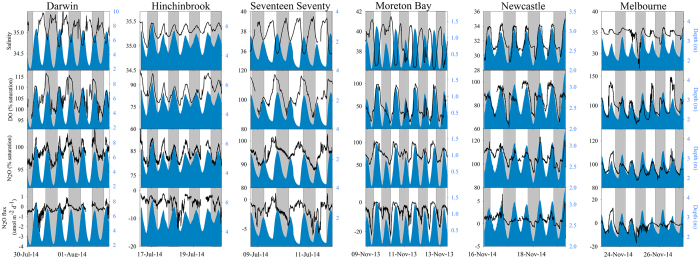
Time series observations in the six mangrove tidal creeks. Vertical grey bars represent night time period. Note the different Y axes scales to highlight temporal gradients. Solid blue shaded area represents tidal height. Salinty and depth data were originally reported in Tait et al. (ref. 35).

**Figure 3:**
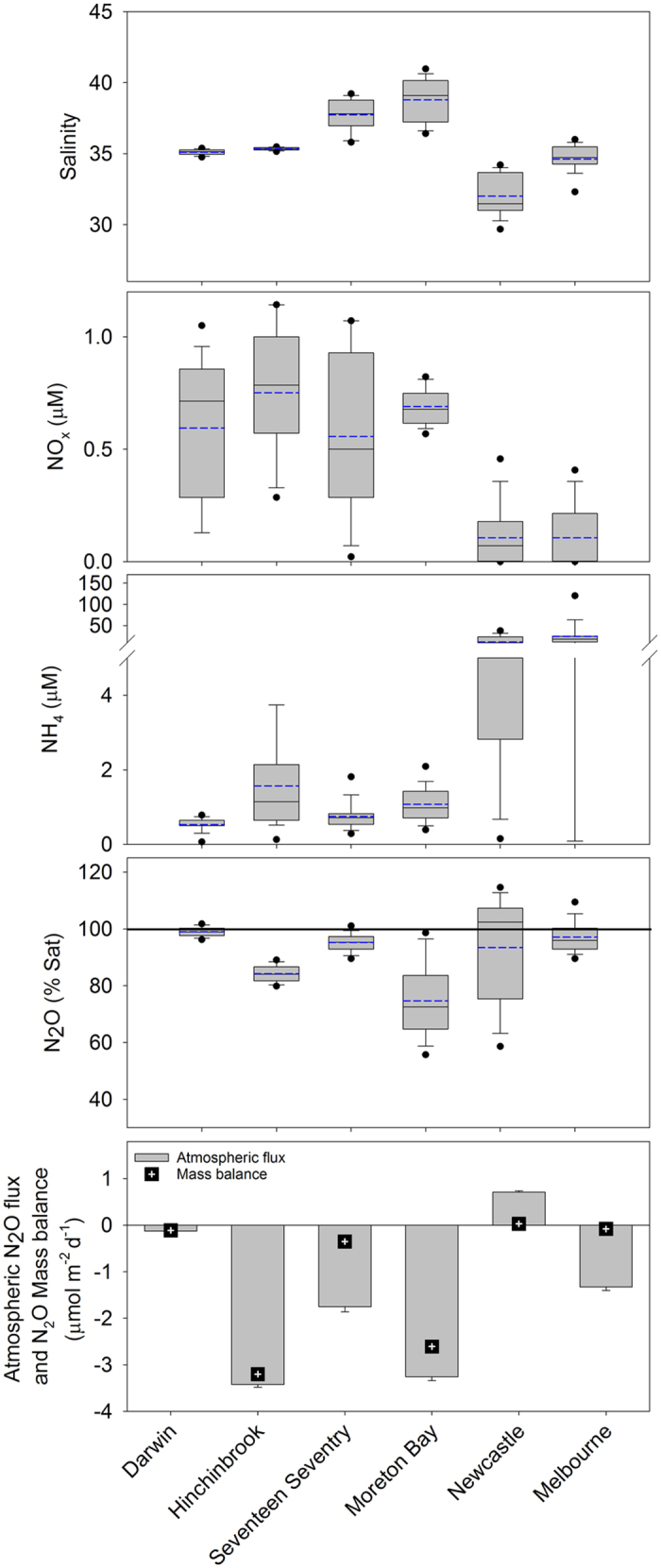
Box plots of salinity, nutrients and N_2_O data from time series observations (5^th^ and 95^th^ percentile, mean = solid black line, median is dashed blue line). Nutrient data for Moreton Bay were from Gleeson *et al*. (ref. 22). Atmospheric N_2_O flux and N_2_O mass balance were mean ±95% confidence interval for each tidal cycle measured, corrected for tidal changes in creek area, and normalised to mangrove intertidal area.

**Figure 4:**
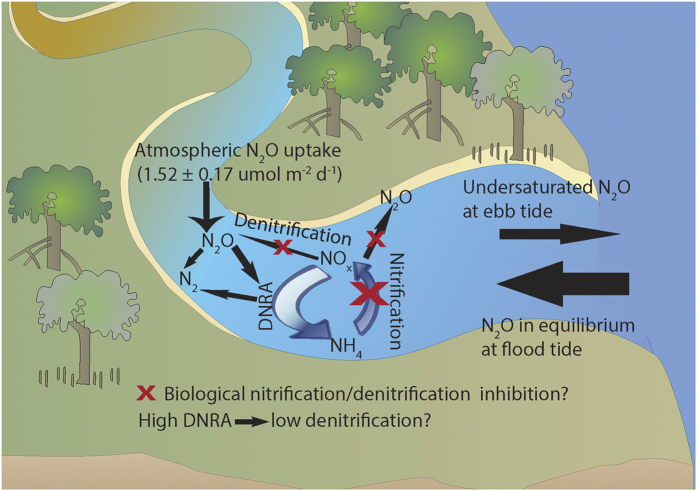
Conceptual model summarizing the potential processes controlling N_2_O dynamics in pristine mangrove creek waters. Figure constructed using symbols courtesy of the Integration and Application Network, University of Maryland Centre for Environmental Science (ian.umces.edu/symbols/).

**Table 1:** Study site characteristics during the 4 to 7 day time series deployments.

	Darwin	HinchinbrookIsland	SeventeenSeventy	MoretonBay	Newcastle	Melbourne
Latitude (°S)	12.4°	18.3°	24.2°	27.8°	32.9°	38.3°
No. of Mangrovespecies	36	29	13	7	3	1
Tide Height						
Max (m)	7.1	3.0	3.6	2.5	1.5	2.1
Min (m)	1.7	0.9	0.7	0.3	0.6	0.4
Range (m)	5.4	2.1	2.9	2.2	0.9	1.7
Average (m)	4.3 ± 1.7	1.8 ± 0.5	1.7 ± 0.8	1.2 ± 0.6	1.0 ± 0.3	1.1 ± 0.4
Air Temperature						
Max (°C)	27.9	23.2	20.0	28.6	26.4	21.2
Min (°C)	25.2	16.4	7.2	19.6	19.8	15.2
Average (°C)	26.5 ± 0.5	21.4 ± 0.7	16.8 ± 2.1	22.9 ± 1.7	23.6 ± 0.9	18.1 ± 1.4
Ave yr temp (°C)	32.0 ± 0.4	28.9 ± 0.5	25.7 ± 0.5	25.2 ± 1.0	21.6 ± 0.6	20.3 ± 0.6
Ave rain (mm yr^−1^)	1729 ± 377	2216 ± 677	1180 ± 326	1336 ± 389	1120 ± 268	621 ± 139
Salinity						
Max	35.5	35.8	39.4	39.5	34.8	36.2
Min	34.7	35.0	35.7	35.4	28.1	27.6
Average	35.1 ± 0.2	35.3 ± 0.1	37.7 ± 1.1	37.0 ± 4.2	31.8 ± 1.6	34.4 ± 1.1
Submerged area						
Max (ha)	72.6	286.3	109.0	37.0	126.6	29.8
Min (ha)	15.9	224.5	3.1	0.8	7.9	0.9
Range (ha)	56.7	61.8	105.9	36.2	118.7	28.9
Average (ha)	57.9 ± 11.1	275.6 ± 10.9	37.2 ± 37.8	25.8 ± 13.9	111.3 ± 31.0	4.8 ± 5.2
Oceanic nutrient concentrations (μM)^1^						
IMOS station	NRSDAR	NRSYON	**	NRSNSI	NRSPHB	NRSMAI
NO_3_	0.43	0.04	0.87	1.69	3.52	2.39
NH_4_	0.22	0.09	0.17	0.24	0.29	0.18

^1^Data are average from nearest IMOS stations over the preceding 5 years, **Data are averaged from stations NRSYON and NRSNSI.

**Table 2:** Field observations of inorganic nitrogen, N_2_O and estimates of air-water fluxes in six pristine Australian mangrove creeks.

		NO_x_ Concentration (μM)		NH_4_ concentration (μM)		N_2_O concentration (nM)		N_2_O saturation (%)		Average atmospheric N_2_O flux^a^ (μmol m^−2^ d^−1^)	Weighted atmospheric N_2_O flux^b^ (μmol m^−2^ d^−1^)	N_2_O mass balance^c^ (μmol m^−2^ d^−1^)
Site	Latitude	Mean	Range	Mean	Range	Mean	Range	Mean	Range	Mean	Daily integrated	Daily integrated
Darwin	−12.45	0.59	<0.1–1.07	0.53	<0.1–0.79	6.3	6.0–6.8	98.9	94.7–104.3	−0.12 ± 0.01d>	−0.07^d^	−0.11
Hinchinbrook	−18.25	0.75	0.29–1.14	1.56	<0.1–5.93	6.1	5.6–6.9	83.3	75.4–90.5	−3.43 ± 0.06^d^	−3.26^d^	−3.20
Seventeen Seventy	−24.17	0.56	<0.1–1.07	0.75	0.29–1.86	7.7	7.1–8.9	94.3	87.6–106.2	−1.75 ± 0.11^d^	−0.48^d^	−0.35
Moreton Bay	−27.78	ND	ND	ND	ND	5.1	3.4–6.6	77.4	50.3–104.7	−3.19 ± 0.08^d^	−1.52^d^	−2.61
Newcastle	−32.85	0.11	<0.1–0.50	12.69	0.5–40.14	7.5	6.4–8.9	106.4	92.1–123.4	0.71 ± 0.03^d^	0.69^d^	0.03
Melbourne	−38.26	0.11	<0.1–0.42	25.50	<0.1–142.86	7.9	6.9–9.1	96.6	85.9–114.8	−1.33 ± 0.07^d^	−0.12^d^	−0.08
**Average**		0.42	<0.1–1.14	8.20	<0.1–142.86	6.8	3.4–9.1	92.9	50.3–123.4	−1.52 ± 0.17	−0.79 ± 0.57	−1.05 ± 0.59

^a^Per m^2^ water area, ±95% confidence interval ^b^weighted for changes in water area and flux rates over the study period and normalised to total catchment area, ^c^calculated as a function of discharge and dissolved N_2_O concentration integrated at 1 minute intervals, normalised to catchment area, ^d^Flux rates determined using empirical transfer velocity equation of Ho *et al*.^33^.
